# Preparation and corrosion resistance of basic carbonate coating on ZK61M magnesium alloy

**DOI:** 10.1016/j.heliyon.2024.e25587

**Published:** 2024-02-04

**Authors:** Yang Yu, Junge Chen, Le Mi, Aihu Feng, Yun Yu, Fei Xiao

**Affiliations:** a Department of Materials Science, Fudan University, Shanghai, 200433, PR China; b Key Laboratory of Inorganic Coating Materials CAS, Shanghai Institute of Ceramics, Chinese Academy of Sciences, Shanghai, 200050, PR China

**Keywords:** Mg-Zn alloy, Carbonic acid solution, Basic carbonate coating, Corrosion protection, In-situ preparation, Layered hydroxide

## Abstract

In this study, a simple in-situ preparation method for coating on Mg-Zn alloy in a carbonic acid solution was investigated. The formation of a precursor carbonate layer on the alloy surface was observed. As the soaking time increased, the solution gradually turned alkaline, leading to the transformation of the precursor into a basic carbonate coating with a layered hydroxide structure. The corrosion potential (*E*_*corr*_) of the coated samples initially decreased and then increased with increasing the soaking time. After 2 h of soaking, the lowest corrosion potential observed was approximately −1.5105 V. At 12 h, the corrosion potential reached around −1.4645 V, which was comparable to that of the ZK61M magnesium alloy. After 48 h, the corrosion potential was measured to be approximately −1.3507 V.

## 1 Introduction

Magnesium alloys have gained popularity as a replacement for aluminum alloys or titanium alloys in various applications due to their high strength-to-weight ratio, low density, high thermal conductivity, electromagnetic shielding, and excellent machining properties. However, magnesium alloys are prone to corrosion in humid or aqueous environment due to their low standard electrode potential. To mitigate this issue, a protective coating is necessary to be applied on the surface of magnesium alloy.

Various methods can be used to prepare surface coatings on magnesium alloys, including air spraying [1], electroplating [2], anodization [3], chemical vapor deposition [4], physical vapor deposition [5,6], laser/ion or electron beam treatment [7], micro-arc oxidation [8,9], and chemical conversion [10]. Among these methods, layered hydroxide [11,12] films have been found to be the most effective in providing corrosion protection for magnesium alloys. These films have a two-dimensional layered structure and can be loaded with corrosion inhibitors such as vanadate [13], molybdate [14] and tungstate [15]. The inherent ion exchange characteristics [16] of layered hydroxide films allow for the release of corrosion inhibitors while capturing corrosive anions, thereby delaying the occurrence of corrosion and improving the corrosion protection ability of magnesium alloys [17]. Layered hydroxide films, such as Mg-Al layered hydroxide film on AZ91D (Mg-Al alloy) [18] and AS21 (Mg-Al silicon alloy) [19], Mg-Fe layered hydroxide film [20] on Mg-Ca alloy [20], and Mg-Mn layered hydroxide film on pure magnesium [21] were successfully prepared using an environmentally friendly carbonate two-step method. The results [2] demonstrated that the composition and crystal structure of magnesium alloys have a significant impact on the microstructure and corrosion resistance of the film.

ZK61M is a magnesium-zinc-zirconium alloy that is widely utilized due to its high strength, excellent plasticity, and corrosion resistance. While previous research has primarily focused on microarc oxidation coatings and electroplated nickel coatings for corrosion protection on ZK61M surfaces, there is a lack of studies on the in-situ growth of layered hydroxide films specifically for this alloy. Therefore, it is of significant importance to investigate the preparation of layered hydroxide coatings on the surface of ZK61M alloy. In this study, the focus was on films loaded with CO32- ions, examining the impact of reaction conditions on the in-situ growth process, film structure, and corrosion resistance. ZK61M is a magnesium-zinc-zirconium alloy that is widely utilized due to its high strength, excellent plasticity and corrosion resistance. While previous research has primarily focused on microarc oxidation coatings and electroplated nickel coatings for corrosion protection on ZK61M surfaces [[22], [23], [24]], there is a lack of studies on the in-situ growth of layered hydroxide films specifically for this alloy.-. Therefore, it is of significant importance to investigate the preparation of layered hydroxide coatings on the surface of ZK61M alloy One crucial aspect in studying corrosion is the measurement of corrosion potential. Corrosion potential refers to the voltage difference between a metal surface and its surrounding environment, which determines the likelihood and rate of corrosion. By assessing the corrosion potential, valuable insights can be gained regarding the corrosive behavior of materials and the prediction of their deterioration over time.

In this study, magnesium-zinc basic carbonate coating with a layered structure as LDH was in-situ prepared on the surface of the Mg-Zn alloy system. The coating exhibits good corrosion resistance and the preparation method is simple and environmentally friendly, making it suitable for large-scale application.

## 2 Experimental

In this study, the ZK61M magnesium alloy was utilized. The alloy conposition consists of 93.88 wt% Mg, 5.43 wt% Zn, 0.65 wt% Zr, and trace amounts (less than 0.01 wt%) of other elements. Square coupon samples were prepared with dimensions of 20*20 mm^2^ and a thickness of 3 mm. The samples underwent grinding using 120 grit, 500 grit, and 1000 grit sandpapers sequentially, followed by cleaning in ethyl alcohol using an ultrasonic cleaner.

A carbonic acid solution was prepared by bubbling CO_2_ gas into 1000 ml of deionized water at 50 °C. The flow rate of CO_2_ gas was maintained at 1 dm^3^/min and after 20 min the pH of the solution reached a range of 4.1–4.3.

The prepared samples were suspended and soaked in the 50 °C carbonated solution for varying durations, ranging from 1 to 48 h. After removal from the solution, the samples were dried in an oven at 50 °C. Each sample was labeled according to its soaking time, suchas MZ-1 (soaked for 1 h), MZ-2 (soaked for 2 h), MZ-36 (soaking for 36 h), MZ-48 (soaking for 48 h).

The surface morphology of the chemical conversion coatings was observed using a field emission scanning electron microscope (SU8220). The cross-sectional morphology was examined using a Focused Ion Beam/Electron Beam Dual Beam Microanalysis System (FEI Versa 3D). The crystallographic structure of the coatings was analyzed using a high-resolution X-ray diffractometer (Bruker D8 Discover) with Cu targetand a scanning range of 5–80° and a speed of 15°/min. The elemental composition of the coatings was analyzed using a XPS X-ray energy spectrometer (Escalab250Xi). The FTIR spectrum of the coatings was obtained using a PerkinElmer Spotlight 400 instrument in the wavenumber range from 400 to 4000 cm^−1^ at room temperature.

The electrochemical polarization measurements of the samples were performed using a 1287 + 1260 electrochemical workstation (AMETEK) with a three-electrode cell setup. A platinum mesh served as the counter electrode, while a silver/silver chloride (Ag/AgCl) electrode (saturated KCl) acted as the reference electrode. The corrosion cell contained a 3.5 wt% NaCl solution at room temperature. The tested sample was used as the working electrode, with an exposed surface area of 1 cm^2^. The corrosion potential and corrosion current density were determined using the Tafel extrapolation method at a scan rate of 1 mVs^-1^. The sine wave excitation signal had an amplitude of 10 mV/s, and the frequency range was 10 mHz-100kHz, with the test potential set as the open circuit potential of the working electrode.

## 3 Results and analysis

### 3.1 Chemical conversion coating

**Fig. 1:**
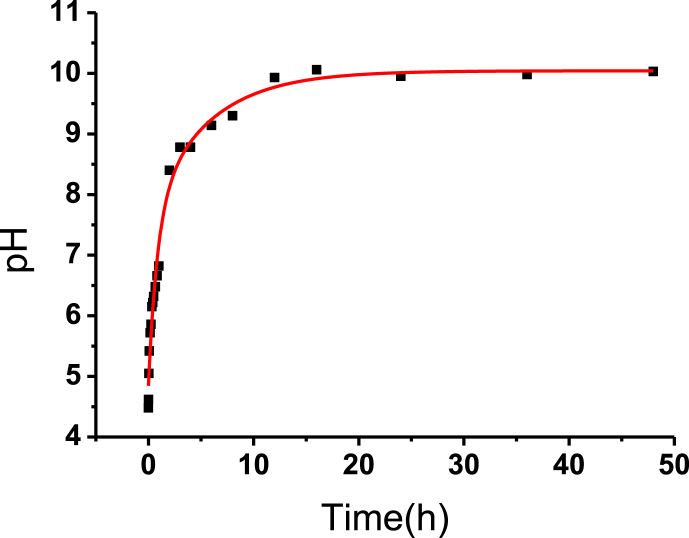
Relationship between pH of solution and time over 48 h.

**Fig. 2:**
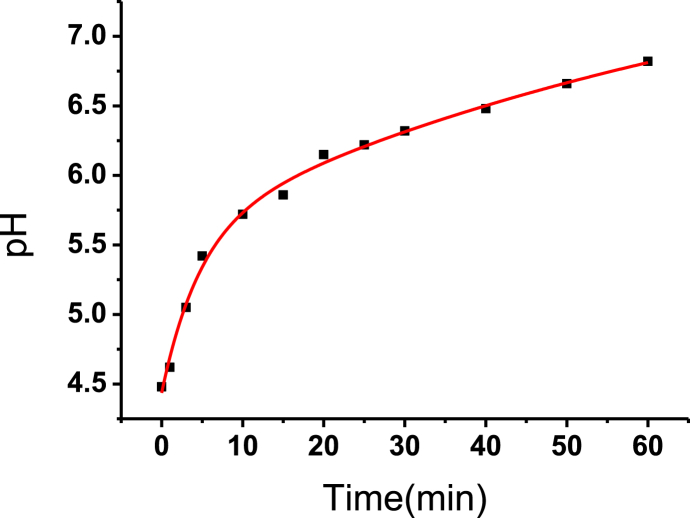
Relationship between pH of solution and time over 1 h.

The pH of the carbonic acid solution exhibited a rapid increase upon addition of the samples, as depicted in Fig. 1. Within the first hour(see Fig. 2), the pH escalated to 6.82 followed by further increments to 8.40 after 2 h, and 9.93 after 12 h. Thereafter, the pH level stabilized at approximately 10 for the duration of the treatment process.

When the ZK61M magnesium alloy samples were corroded in carbonic acid solution, the cathode reaction was:

(1) 2e^−^ + 2H^+^(aq) → H_2_(g)

The anode reaction was:

(2) Mg(s) − 2e^−^ → Mg^2+^(aq), Zn(s) − 2e^−^ → Zn^2+^(aq)

As the reaction progressed, H^+^ ions were consumed, causing the pH of the solution to rise rapidly. The solution gradually transitioned from weakly acidic to neutral. The following reactions took place in the solution:

(3) Zn^2+^(aq) + CO_3_^2−^(aq) → ZnCO_3_(s)

(4) Mg^2+^(aq) + CO_3_^2−^(aq) → MgCO_3_(s)

As the samples continued to react in a neutral solution, the cathode reaction was:

(5) 2H_2_O(aq) + 2e^−^ → 2OH^−^(aq) + H_2_(g)

The anode reactions remained the same:

(6) Mg(s) − 2e^−^ → Mg^2+^(aq), Zn(s) − 2e^−^ → Zn^2+^(aq)

As the reaction continued, a large amount of OH^−^ ions were generated, causing the pH of the solution to rise and eventually reach equilibrium around pH 10. The solution gradually shifted from neutral to weakly basic.

The following reactions occurredin the solution:

(7) Zn^2+^(aq) + CO_3_^2−^(aq) → ZnCO_3_(s)

(8) Mg^2+^(aq) + CO_3_^2−^(aq) → MgCO_3_(s)

(9) ZnCO_3_(s) + OH^−^(aq) → Zn(OH)_2_(s)

(10) MgCO_3_(s) + OH^−^(aq) → Mg(OH)_2_(s)

Therefore, the overall corrosion reaction of the ZK61M magnesium alloy samples in the carbonic acid solution can be represented as:

(11) Mg(s) + Zn(s) + H_2_CO_3_(aq) + H_2_O(aq) → Mg/Zn/(OH)/CO_3_⋅H_2_O + H_2_(g)

This resulted in the formation of a base carbonate on the surface of the ZK61M magnesium alloy samples.

### 3.2 Microstructure

**Fig. 3:**
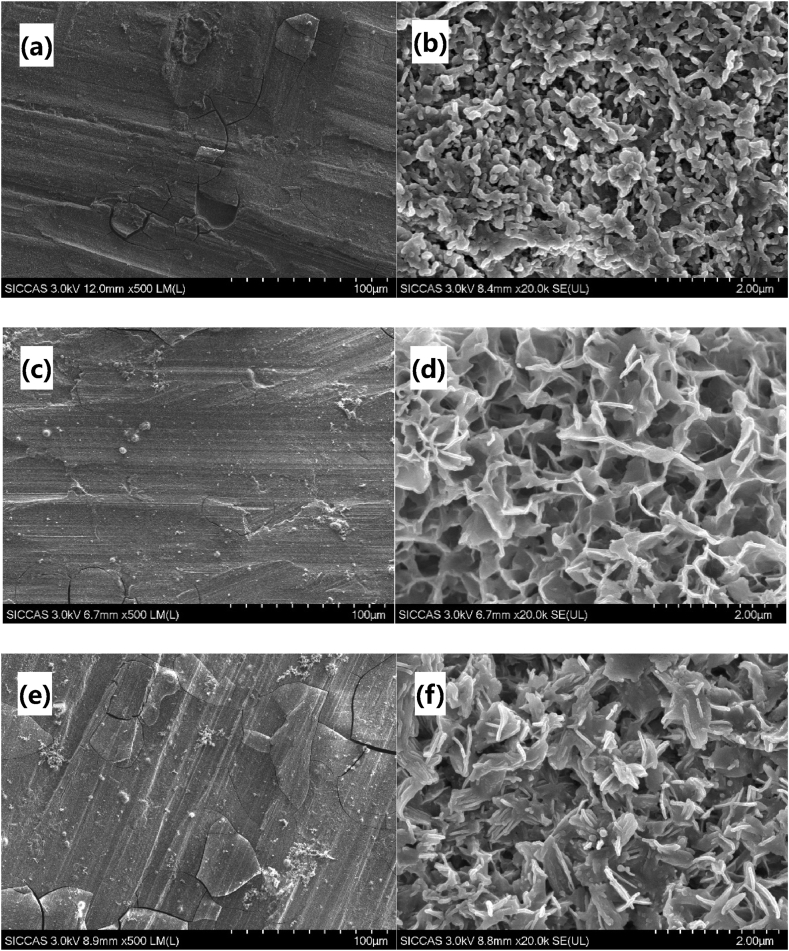
Surface morphology of the coatings at different reaction times (a) MZ-1; (b) MZ-1; (c) MZ-24; (d) MZ-24; (e) MZ-48; (f) MZ-48.

The photographs in Fig. 3 depict the surface morphology of the samples after reacting in the carbonate solution for different durations: 1 h, 24 h and 48 h. In Fig. 3(a), (c), and (e), it can be observed that complete coatings have formed on the sample surfaces. However, there are varying degrees of cracking, which are typical characteristics of chemical conversion films. Further examination at higher magnification reveals that the coating on the MZ-1 sample exhibits a rod-like structure with a diameter of approximately 100 nm (Fig. 3(b)). The coating on the MZ-24 sample displays a distinct flake network structure with a flake thickness of around 50 nm (Fig. 3(d)). The coating on MZ-48 sample exhibits a lamellar structure with evident layered hydroxide characteristics, and the flake thickness is approximately 50 nm (Fig. 3(f)). These findings indicate that the reaction time significantly influences the growth of the surface coating on the samples [10].

**Fig. 4:**
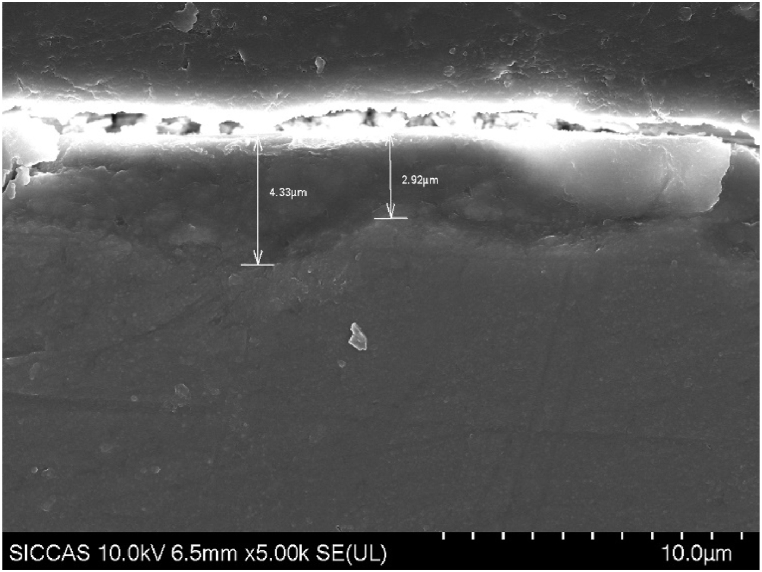
Cross-sectional surface morphology of the sample observed using SEM.

The microstructure of the sample's cross-section is shown in Fig. 4. A uniform reaction layer, with an average thickness of about 4 μm, can be observed on the sample's surface of the sample with an average thickness of about 4 μm.

**Fig. 5:**
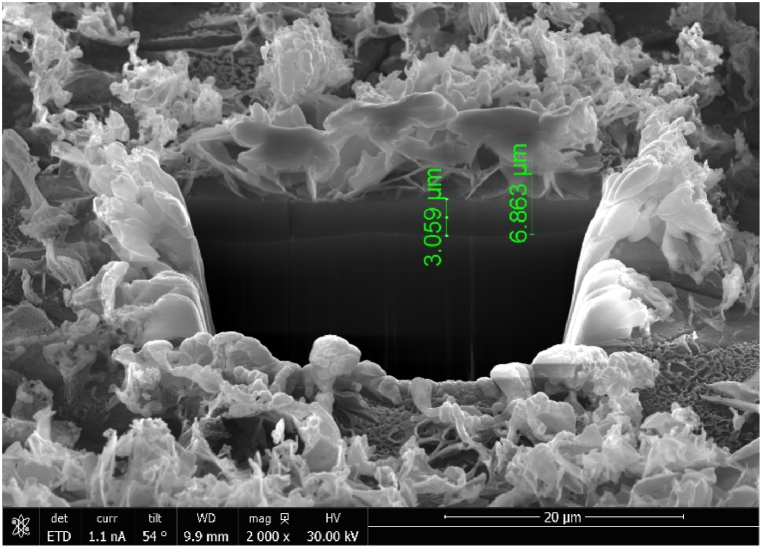
Cross-sectional surface morphology of the sample observed using FIB/SEM.

**Fig. 6:**
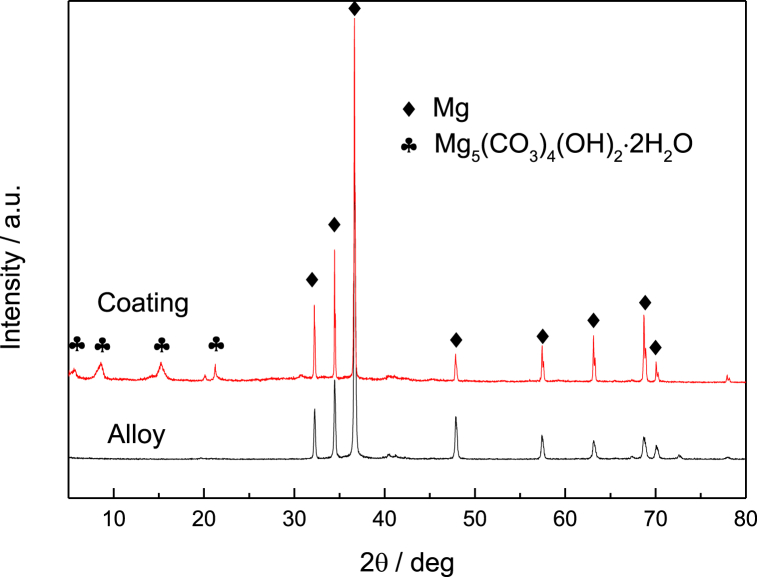
XRD patterns of the coating and ZK61M magnesium alloy.

The cross-sectional structure of the sample after focused ion beam cutting is depicted in Fig. 5. It can be clearly seen that the coating is divided into two layers. One layer is a dense coating structure with a thickness of about 3 μm, while the other layer is a loose, lamellar structure with a thickness of about 4 μm. XRD tests were conducted to confirm the composition of the material formed on the surface, and the results are presented in Fig. 6. The diffraction peaks of Mg_5_(CO_3_)_4_(OH)_2_⋅2H_2_O (JCPDS No. 23-1218) are observed at 5.6°, 8.6°, 15.2° and 21.2° for the sample with the deposited coating compared to ZK61M magnesium alloy. However, no obvious diffraction peaks of Zn oxide, Zn hydroxide or Zn carbonate diffraction peaks were observed.

**Fig. 7:**
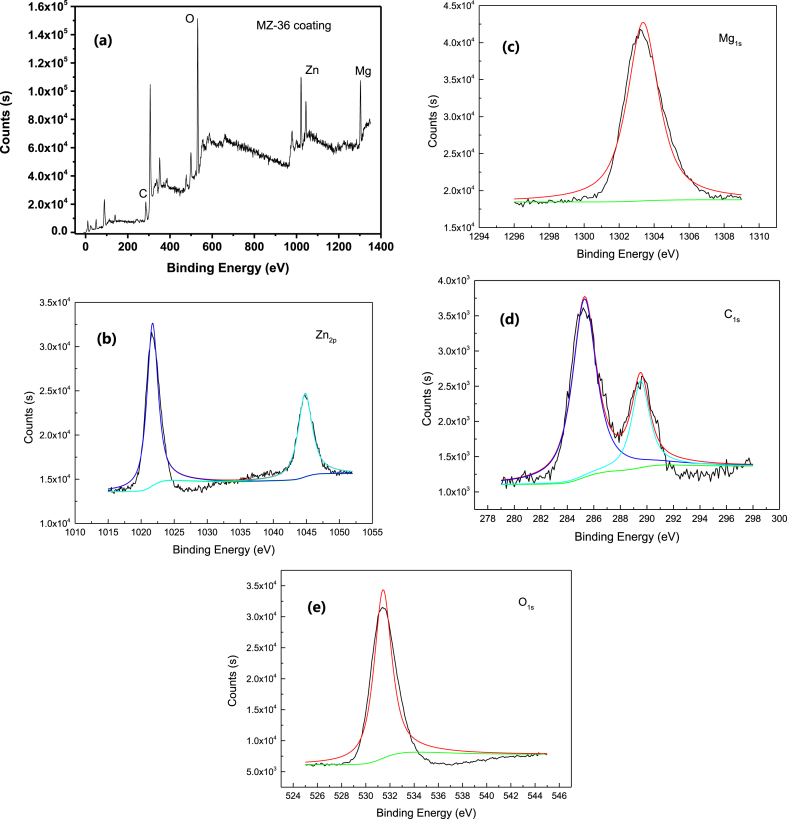
XPS spectra of the coating (MZ-36) (a) survey spectra; High resolution (b) Zn, (c) Mg, (d) C and (e) O peaks acquired by XPS.

Fig. 7(a) displays the XPS spectras [25,26] of the coating elements on the sample's surface. It is evident that Zn, Mg, C and O elements are present in the coating, with the proportion of Mg and Zn in the coating being lower than that in the ZK61M alloy as shown in Table 1. This indicates a greater involvement of Zn elements in the reaction.

**Table 1:** XPS elemental composition of the coating.

Name	Atomic %
C1s	18.54
O1s	48.51
Zn2p	9.27
Mg1s	23.68

The Zn double peaks are located at 1021.72eV and 1044.82eV corresponding to the 2p 3/2 and 2p 1/2 of Zn (Fig. 7(b)), respectively. The distance between the two split orbitals is 23.10eV, suggesting the presence of Zn^2+^ ions in the coating. This further confirms the dissolution of Zn elements from the ZK61M magnesium alloy and their involvement in the coating formation. The peak of Mg is located at 1303.37eV, corresponding to the 1s state of Mg (Fig. 7(c)). The peaks of C are located at 285.29eV and 289.53eV, corresponding to the 1s and MCO_3_ states of C (Fig. 7(c)), respectively, indicating the presence of carbonates in the coating. The peak of O is located at 531.43eV, corresponding to the 1s state of O (Fig. 7(e)).

**Fig. 8:**
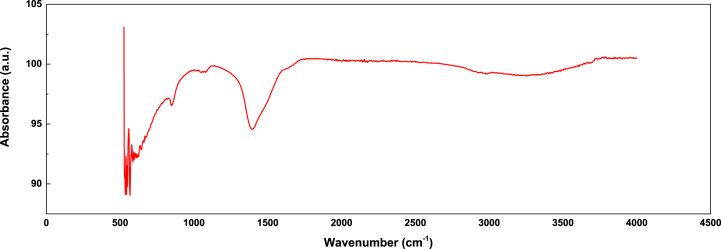
FTIR spectrum of the coating on the MZ-24 sample.

The FT-IR spectra of the coating on the MZ-24 sample is shown in Fig. 8. The wide absorption band at ∼3248 cm^−1^ is attributed to the O-H stretching vibration in Zn(OH)_2_. The absorption band at ∼1396 cm^−1^ corresponds to the asymmetric stretching vibration of CO_3_^−^ in carbonate. The band at ∼840 cm^−1^ is assigned to the out-of-plane bending vibration of CO_3_^2−^. The peak at 621 cm^−1^ corresponds to the in-plane bending vibration of CO_3_^2−^. The peaks at 550 cm^−1^ and 614 cm^−1^ can be attributed to the Mg-O vibration in Mg(OH)_2_ [27]. Based on the XPS spectra and FTIR spectrum, it can be concluded that a basic carbonate coating of magnesium and zinc was successfully prepared on the surface of ZK61M magnesium alloy.

### 3.3 Anti-corrosion property

**Fig. 9:**
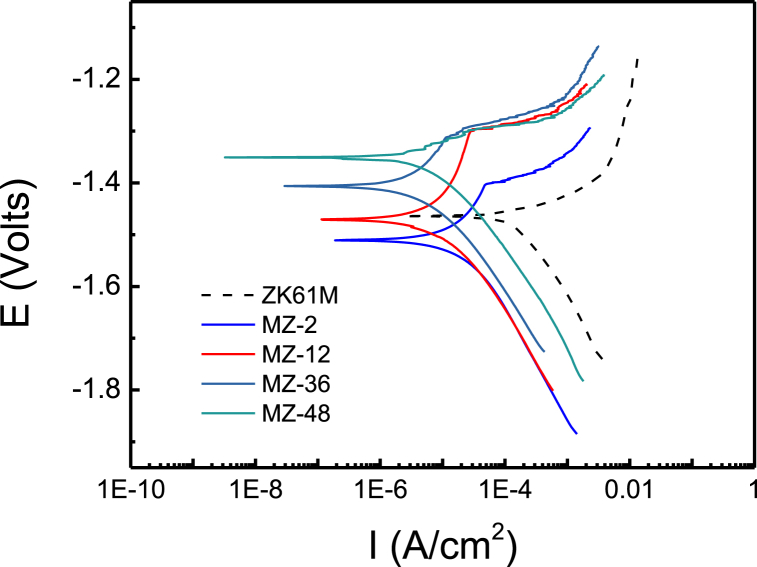
Polarization curves of the ZK61M magnesium alloy, MZ-2, MZ-12, MZ-36 and MZ-48. The electrochemical tests were performed in 3.5 wt% NaCl solution.

**Fig. 10:**
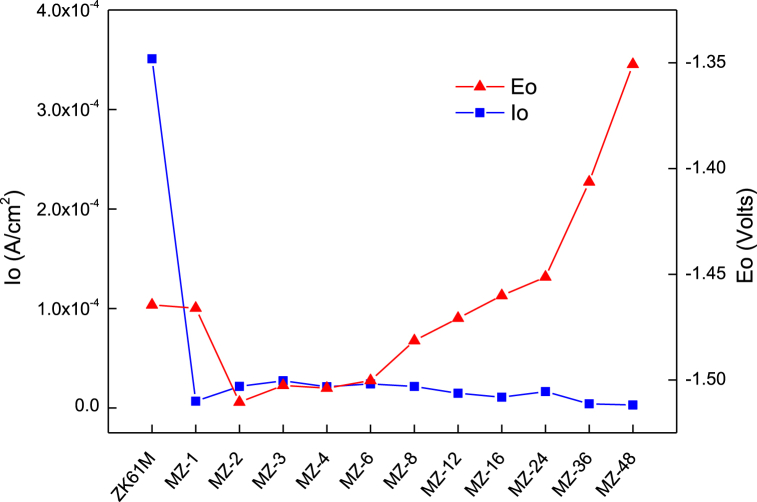
Corrosion potential and corrosion current density of the test samples.

Fig. 9 illustrates the potentiodynamic polarization curves of ZK61M magnesium alloy and its samples immersed in carbonic acid solution for different durations, ranging from 2 h to 48 h, in 3.5 % NaCl solution. Fig. 10 presents the statistics of corrosion current and corrosion potential density. From Fig. 9, it can be observed that the polarization curves of ZK61M magnesium alloy and the samples with different immersion times are similar, indicating that they are all controlled by electrochemical polarization. Fig. 10 shows that with increasing immersion time, the corrosion potential initially decreases and then increases, while the corrosion current density exhibits a trend of first increases and then decreases after a sharp decrease.

The corrosion potential of MZ-2 samples, immersed in a carbonic acid solution for 2 h was the lowest at approximately −1.5105 V, which was 0.046 V lower than that of ZK61M magnesium alloy (−1.4645 V). The corrosion potential of MZ-48 samples, immersed in a carbonic acid solution for 48 h, was the highest at about −1.3507 V, which was approximately 0.114 V higher than that of ZK61M magnesium alloy. The corrosion current density of MZ-1 samples, immersed in a carbonic acid solution for 1 h was approximately 6.75 × 10^−6^A/cm^−2^, which is approximately 2 % of ZK61M magnesium alloy (3.51 × 10^−4^A/cm^−2^). The corrosion current density of MZ-48 samples, immersed in a carbonic acid solution for 48 h was the lowest at about 2.88 × 10^−6^A/cm^−2^, which is approximately 0.8 % of ZK61M magnesium alloy. Based on the above analysis, it is believed that ZK61M magnesium alloy initially reacts with carbonic acid in the solution, forming carbonate products with a porous and loose structure. This weakens the corrosion resistance of the surface coating of ZK61M magnesium alloy, leading to a decrease in the corrosion potential of the coating. As the reaction progresses, H^+^ ions are consumed, causing the solution to gradually shift from weakly acidic to neutral. The carbonate products formed in the acidic environment gradually transform into basic carbonates with a denser structure in the neutral environment. Consequently, the corrosion resistance of the surface coating of ZK61M magnesium alloy gradually improves. After 2 h of soaking, the corrosion potential of the coating starts to increase as the solution becomes weakly alkaline. After 12 h of soaking, the pH value of the solution reaches approximately 10, and the corrosion potential of the coating becomes approximately −1.4708 V, which is essentially the same as that of ZK61M magnesium alloy (−1.4645 V). With increasing soaking time, the pH value of the solution stabilizes at approximately 10, and the corrosion potential of the coating continued to increase at accelerated rate. This indicates that more and more carbonate products are transforming into dense basic carbonates thereby further improving surface density of the coating. After 48 h of soaking, the corrosion potential of the coating reaches its highest value, and the corrosion current density reached the lowest value. Therefore, the coating exhibits the best anti-corrosion effect.

**Fig. 11:**
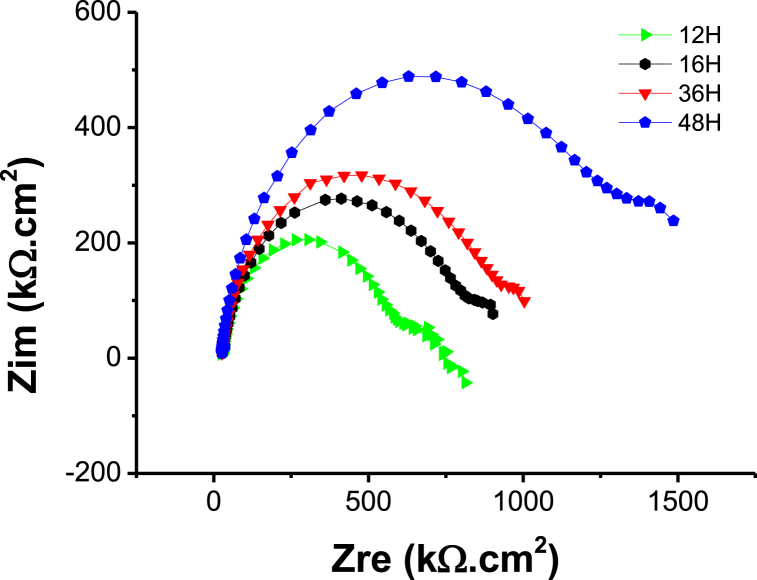
Impedance spectra of coatings.

Fig. 11 illustrates the electrochemical impedance spectra (EIS) of ZK61M magnesium alloy samples immersed in 3.5 % NaCl solution. The samples were exposed to carbonic acid solution for different soaking times, ranging from 12 h and beyond. At least 5 sets of samples were tested, and the test results of each set of samples were very similar. Upon observing Fig. 11, it can be noted that once the pH value of the reaction system stabilizes, the Nyquist plots of the samples exhibit similar characteristics. These plots consist of three distinct regions: a high frequency capacitive arc, a medium-frequency capacitive arc and a low-frequency inductive arc. The size of the capacitive arc corresponds to the polarization resistance of the surface coating. A larger arc radius indicates greater polarization resistance, which implies better corrosion resistance and slower corrosion rate. The high-frequency -and -middle-frequency capacitive arcs likely relate to the charge transfer process and the formation of a corrosion product layer facilitated by the presence of coating. The low-frequency inductive arc is attributed to the dissolution of ZK61M magnesium alloy and the detachment of the coating. The Nyquist plot of Fig. 11 demonstrates that as the immersion time increases, the high-frequency capacitive arc of the electrochemical impedance spectra exhibits a significant growth. This indicates that prolonging the immersion time effectively enhances the corrosion resistance of the coating.

**Fig. 12:**
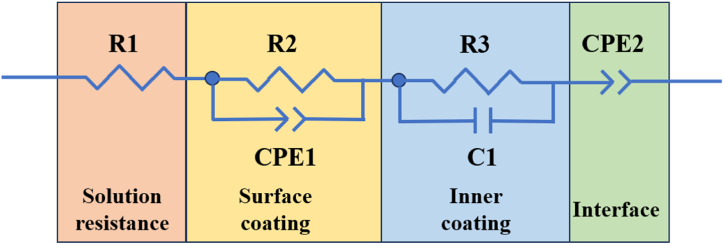
Equivalent circuit model for the impedance spectrum of the samples.

The impedance spectrum was simulated using Zview 3.5 software to obtain the EIS equivalent circuit model of the samples subjected to soaking treatment, as depicted in Fig. 12. From the figure, it can be observed that the equivalent circuit model contains of four components. R1 represents the resistance of the solution, while R2 and CPE1 represent the polarization resistance and inductance of the surface compound respectively. Additionally, R3 and C1 account for the polarization resistance and capacitance of the inner coating respectively. The presence of an inductive resistance phenomenon of CPE2 suggests that charge migration and adsorption reactions take place at the interface between the coating and the metal.

Based on the findings of this study, the method of preparing alkaline carbonate coatings on Mg-Zn alloy shows great potential for large-scale production. It is recommended to maintain a pH range of 9–10 for the coating solution, as it has been shown to effectively promote the formation of alkaline carbonate coatings with desirable properties. Immersing the samples for 24–48 h is suggested to ensure the formation of a dense and corrosion-resistant alkaline carbonate coating. It is advisable to maintain the reaction temperature at around 50 °C to avoid any potential adverse effects on the coating formation. By following these recommendations, the large-scale production of alkaline carbonate coatings on Mg-Zn alloy can be easily achieved. This method offers a pollution-free and straightforward approach to enhance the corrosion resistance of Mg-Zn alloy, making it suitable for various industrial applications.

## 4 Conclusion

1) A basic carbonate film, Mg_5_(CO_3_)_4_(OH)_2_⋅2H_2_O, with a layered hydroxide structure was formed on the surface of ZK61M magnesium alloy using an aqueous CO_2_ solution.
2) As time increases, the pH value of the system also increases, which promotes the deposition of basic carbonate. After 48 h of soaking, the film layer exhibits a distinct layered hydroxide structure, and is relatively dense and complete.

The basic carbonate film prepared using the CO_2_ aqueous solution effectively enhances the corrosion resistance of ZK61M magnesium alloy. The MZ-48 sample immersed in carbonic acid solution for 48 h exhibited the highest corrosion potential of approximately −1.3507 V. The corrosion rate (corrosion current density) in 3.5 % NaCl solution was 2.88 × 10^−6^A/cm^−2^, which is about 0.8 % of the corrosion rate of ZK61M magnesium alloy (3.51 × 10^−4^A/cm^−2^). The polarization impedance is approximately 490 kΩ cm^2^.

## Data availability statement

The authors confirm that the data supporting the findings of this study are available within the article.

## Additional information

No additional information is available for this paper.

## CRediT authorship contribution statement

**Yang Yu:** Writing – review & editing, Writing – original draft, Validation, Supervision, Resources, Project administration, Methodology, Investigation, Funding acquisition, Conceptualization. **Junge Chen:** Validation, Data curation. **Le Mi:** Validation, Methodology. **Aihu Feng:** Validation, Data curation. **Yun Yu:** Writing – review & editing, Methodology, Funding acquisition, Conceptualization. **Fei Xiao:** Writing – review & editing, Methodology, Conceptualization.

## Declaration of competing interest

The authors declare that they have no known competing financial interests or personal relationships that could have appeared to influence the work reported in this paper.
